# Supplementary material for: The validity and reliability of the Dutch version of the Student Satisfaction and Self-Confidence in Learning Scale (SCLC) for pharmacy technicians
Source: PLoS One. 2025 Sep 29;20(9):e0331115. doi: 10.1371/journal.pone.0331115 (PMC12478918; doi:10.1371/journal.pone.0331115)
Supplement: S1 Table — This table presents Pearson correlation coefficients between individual items of the SCLC questionnaire. Significant correlations are denoted by p < 0.05 (*), p < 0.01 (**). High correlations can be observed between items such as ITEM 7 and ITEM 8 (r = 0.724), and ITEM 4 and ITEM 5 (r = 0.658**), suggesting strong relationships within certain subscales. ITEM 13R, a reverse-coded item, generally shows weaker or negative correlations with other items. This table illustrates the internal consistency and inter-item relationships across the questionnaire. (DOCX) [file pone.0331115.s001.docx]

| **Items** | **ITEM 1** | **ITEM 2** | **ITEM 3** | **ITEM 4** | **ITEM 5** | **ITEM 6** | **ITEM 7** | **ITEM 8** | **ITEM 9** | **ITEM 10** | **ITEM 11** | **ITEM 12** | **ITEM 13R** |
| --- | --- | --- | --- | --- | --- | --- | --- | --- | --- | --- | --- | --- | --- |
| **ITEM 1** | 1.000 | 0.595** | 0.440** | 0.478** | 0.426** | 0.356** | 0.490** | 0.516** | 0.385** | 0.427** | 0.266** | 0.537** | 0.058 |
| **ITEM 2** | 0.595** | 1.000 | 0.448** | 0.504** | 0.311** | 0.255** | 0.527** | 0.559** | 0.382** | 0.362** | 0.267** | 0.521** | -0.073 |
| **ITEM 3** | 0.440** | 0.448** | 1.000 | 0.520** | 0.451** | 0.405** | 0.238** | 0.410** | 0.533** | 0.351** | 0.415** | 0.408** | -0.066 |
| **ITEM 4** | 0.478** | 0.504** | 0.520** | 1.000 | 0.658** | 0.451** | 0.529** | 0.658** | 0.539** | 0.347** | 0.282** | 0.471** | 0.013 |
| **ITEM 5** | 0.426** | 0.311** | 0.451** | 0.658** | 1.000 | 0.474** | 0.380** | 0.536** | 0.514** | 0.144 | 0.214* | 0.292** | 0.003 |
| **ITEM 6** | 0.356** | 0.255** | 0.405** | 0.451** | 0.474** | 1.000 | 0.421** | 0.595** | 0.436** | 0.391** | 0.360** | 0.443** | 0.168 |
| **ITEM 7** | 0.490** | 0.527** | 0.238** | 0.529** | 0.380** | 0.421** | 1.000 | 0.724** | 0.393** | 0.151 | 0.335** | 0.580** | -0.036 |
| **ITEM 8** | 0.516** | 0.559** | 0.410** | 0.658** | 0.536** | 0.595** | 0.724** | 1.000 | 0.582** | 0.295** | 0.472** | 0.647** | 0.138 |
| **ITEM 9** | 0.385** | 0.382** | 0.533** | 0.539** | 0.514** | 0.436** | 0.393** | 0.582** | 1.000 | 0.349** | 0.334** | 0.374** | 0.043 |
| **ITEM 10** | 0.427** | 0.362** | 0.351** | 0.347** | 0.144 | 0.391** | 0.151 | 0.295** | 0.349** | 1.000 | 0.335** | 0.400** | 0.132 |
| **ITEM 11** | 0.266** | 0.267** | 0.415** | 0.282** | 0.214* | 0.360** | 0.335** | 0.472** | 0.334** | 0.335** | 1.000 | 0.447** | -0.082 |
| **ITEM 12** | 0.537** | 0.521** | 0.408** | 0.471** | 0.292** | 0.443** | 0.580** | 0.647** | 0.374** | 0.400** | 0.447** | 1.000 | 0.102 |
| **ITEM 13R** | 0.058 | -0.073 | -0.066 | 0.013 | 0.003 | 0.168 | -0.036 | 0.138 | 0.043 | 0.132 | -0.082 | 0.102 | 1.000 |
