# Supplementary material for: The validity and reliability of the Dutch version of the Student Satisfaction and Self-Confidence in Learning Scale (SCLC) for pharmacy technicians
Source: PLoS One. 2025 Sep 29;20(9):e0331115. doi: 10.1371/journal.pone.0331115 (PMC12478918; doi:10.1371/journal.pone.0331115)
Supplement: S3 Table — CR and AVE are calculated for the full 13-item model without adjustments. (DOCX) [file pone.0331115.s003.docx]

| **Factor** | **Composite Reliability (CR)** | **Average Variance Extracted (AVE)** |
| --- | --- | --- |
| **Satisfaction** | 0.824 | 0.486 |
| **Self-confidence** | 0.804 | 0.399 |
