# Supplementary material for: The validity and reliability of the Dutch version of the Student Satisfaction and Self-Confidence in Learning Scale (SCLC) for pharmacy technicians
Source: PLoS One. 2025 Sep 29;20(9):e0331115. doi: 10.1371/journal.pone.0331115 (PMC12478918; doi:10.1371/journal.pone.0331115)
Supplement: S4 Table — This table presents the indices of goodness of fit for the model, alongside reference values for a good model fit in the right-hand column. The indices include the Comparative Fit Index (CFI), Goodness of Fit Index (GFI), Adjusted Goodness of Fit Index (AGFI), Root Mean Square Error of Approximation (RMSEA), and Root Mean Square Residual (RMR), with values compared to standard thresholds. The chi-square test for goodness of fit and the chi-square to degrees of freedom ratio (χ2/df) are also reported. Fit-indices are calculated where ITEM 10 and ITEM 13 are removed- without MI-based modifications and without correlated error terms. (DOCX) [file pone.0331115.s004.docx]

| Index | Value | Values for good model fit |
| --- | --- | --- |
| CFI | 0.89 | >0.97 (>0.9 is traditional) |
| GFI | 0.84 | >0.95 |
| AGFI | 0.75 | >0.80 |
| RMSEA | 0.119 | <0.08 good; 0.5-.10 moderate |
| RMR | 0.048 | <0.05 |
| Goodness of fit test | χ2 =120.060; df=43; p < 0.000 |  |
| Reason for fit | χ2/ gl = 2.79; | Between 1-3 |
