# Supplementary figures and images for: The validity and reliability of the Dutch version of the Student Satisfaction and Self-Confidence in Learning Scale (SCLC) for pharmacy technicians
Source: PLoS One. 2025 Sep 29;20(9):e0331115. doi: 10.1371/journal.pone.0331115 (PMC12478918; doi:10.1371/journal.pone.0331115)

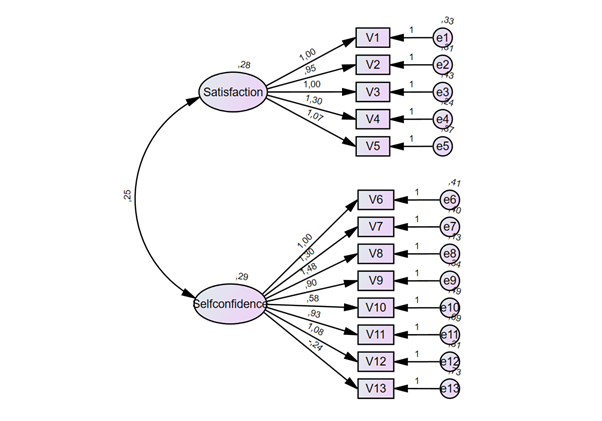

Supplement: S6 Fig — No MI-based error correlations are made. (TIF) [file pone.0331115.s006.tif]

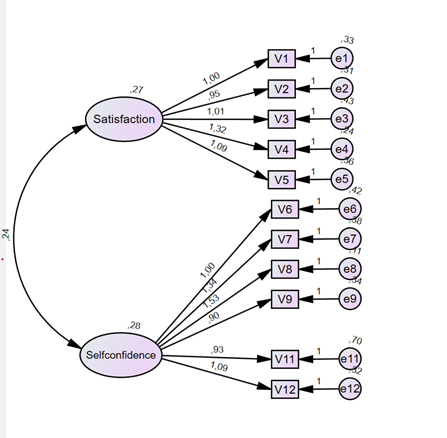

Supplement: S7 Fig — No MI- based error correlations are made. (TIF) [file pone.0331115.s007.tif]
