# Supplementary material for: The validity and reliability of the Dutch version of the Student Satisfaction and Self-Confidence in Learning Scale (SCLC) for pharmacy technicians
Source: PLoS One. 2025 Sep 29;20(9):e0331115. doi: 10.1371/journal.pone.0331115 (PMC12478918; doi:10.1371/journal.pone.0331115)
Supplement: S8 File — Available from the National League for Nursing. The questionnaire is owned and copyrighted by the National League for Nursing. (PDF) [file pone.0331115.s008.pdf]

Student Satisfaction and Self-Confidence in Learning

Instructions: This questionnaire is a series of statements about your personal attitudes about the instruction you receive during your simulation activity. Each item represents a statement about your attitude toward your satisfaction with learning and self-confidence in obtaining the instruction you need. There are no right or wrong answers. You will probably agree with some of the statements and disagree with others. Please indicate your own personal feelings about each statement below by marking the numbers that best describe your attitude or beliefs. Please be truthful and describe your attitude as it really is, not what you would like for it to be. This is anonymous with the results being compiled as a group, not individually.

Mark:
- 1 = STRONGLY DISAGREE with the statement
- 2 = DISAGREE with the statement
- 3 = UNDECIDED - you neither agree or disagree with the statement
- 4 = AGREE with the statement
- 5 = STRONGLY AGREE with the statement

**Satisfaction with Current Learning**	**SD**	**D**	**UN**	**A**	**SA**
1. The teaching methods used in this simulation were helpful and effective.	○ 1	○ 2	○ 3	○ 4	○ 5
2. The simulation provided me with a variety of learning materials and activities to promote my learning the medical surgical curriculum.	○ 1	○ 2	○ 3	○ 4	○ 5
3. I enjoyed how my instructor taught the simulation.	○ 1	○ 2	○ 3	○ 4	○ 5
4. The teaching materials used in this simulation were motivating and helped me to learn.	○ 1	○ 2	○ 3	○ 4	○ 5
5. The way my instructor(s) taught the simulation was suitable to the way I learn.	○ 1	○ 2	○ 3	○ 4	○ 5
**Self-confidence in Learning**	**SD**	**D**	**UN**	**A**	**SA**
6. I am confident that I am mastering the content of the simulation activity that my instructors presented to me.	○ 1	○ 2	○ 3	○ 4	○ 5
7. I am confident that this simulation covered critical content necessary for the mastery of medical surgical curriculum.	○ 1	○ 2	○ 3	○ 4	○ 5
8. I am confident that I am developing the skills and obtaining the required knowledge from this simulation to perform necessary tasks in a clinical setting	○ 1	○ 2	○ 3	○ 4	○ 5
9. My instructors used helpful resources to teach the simulation.	○ 1	○ 2	○ 3	○ 4	○ 5
10. It is my responsibility as the student to learn what I need to know from this simulation activity.	○ 1	○ 2	○ 3	○ 4	○ 5
11. I know how to get help when I do not understand the concepts covered in the simulation.	○ 1	○ 2	○ 3	○ 4	○ 5
12. I know how to use simulation activities to learn critical aspects of these skills.	○ 1	○ 2	○ 3	○ 4	○ 5
13. It is the instructor's responsibility to tell me what I need to learn of the simulation activity content during class time..	○ 1	○ 2	○ 3	○ 4	○ 5

© Copyright, National League for Nursing, 2005

Revised December 22, 2004
