# Supplementary material for: The validity and reliability of the Dutch version of the Student Satisfaction and Self-Confidence in Learning Scale (SCLC) for pharmacy technicians
Source: PLoS One. 2025 Sep 29;20(9):e0331115. doi: 10.1371/journal.pone.0331115 (PMC12478918; doi:10.1371/journal.pone.0331115)
Supplement: S9 File — (PDF) [file pone.0331115.s009.pdf]

Deze vragenlijst gaat over leren op en voor het werk en hoe jij daarover denkt.
De vragenlijst bestaat uit een reeks stellingen over jouw persoonlijke houding ten opzichte van de alle leeractiviteiten die je hebt gehad.

Leeractiviteiten zijn alle manieren die je op het Erasmus MC hebt gehad om te leren. Denk hierbij aan e-modules (CME-online, Eduplaza, Mediavision, Start Well Stay Excellent (SWSE), klinische lessen (die vroeger koffiepraatjes heetten), het inwerkproces in de apotheek, etc.

Elk item vertegenwoordigt een uitspraak over jouw houding ten opzichte van jouw tevredenheid met leren en zelfvertrouwen. Er zijn geen goede of foute antwoorden.
Geef bij elke stelling het antwoord dat jouw houding het beste beschrijft.
Wees hierin alsjeblieft eerlijk en geef je gevoel weer zoals het werkelijk is, niet wat je zou willen dat het is. Je antwoorden worden anoniem verwerkt en de resultaten worden voor de hele groep geanalyseerd.

Instructeurs/ docenten zijn mensen die je bijvoorbeeld inwerken of een klinische les verzorgen. In een e-module is een instructeur iemand die de stof uitlegt.

De antwoordopties zijn bij elke vraag hetzelfde:
1. Sterk mee oneens
2. Oneens
3. Neutraal (je bent het dus niet eens, maar ook niet oneens met de stelling)
4. Eens
5. Sterk mee eens

Tevredenheid

1. De leeractiviteiten waren nuttig en effectief
2. De leeractiviteiten boden me een verscheidenheid aan leermaterialen en activiteiten om mijn farmaceutische kennis te vergroten
3. Ik vond de manier waarop het onderwijs werd gegeven leuk
4. De onderwijsmethoden waren motiverend en hebben mij geholpen om te leren
5. De manier waarop ik het onderwijs kreeg, was geschikt voor de manier waarop ik leer

Zelfvertrouwen in het leren

6. Ik heb er vertrouwen in dat ik de inhoud van de leeractiviteiten goed onder de knie heb
7. Ik heb er vertrouwen in dat in de leeractiviteiten die ik heb gehad, belangrijke onderwerpen zijn behandeld, die noodzakelijk zijn om mijn werk in het ziekenhuis goed uit te voeren
8. Ik heb er vertrouwen in dat ik de kennis ontwikkel en de vereiste vaardigheden uit de leeractiviteiten verkrijg voor mijn taken als apothekersassistent
9. De instructeurs/docenten gebruikten nuttige bronnen om mij dingen te leren
10. Het is mijn verantwoordelijkheid als apothekersassistent om te leren wat nodig is van de leeractiviteiten
11. Ik weet waar ik hulp moet vragen, wanneer ik de aangeboden stof niet goed begrijp
12. Ik weet hoe ik leeractiviteiten moet gebruiken om vraagstukken op te lossen

13. Het is de verantwoordelijkheid van de instructeur/docent om mij te vertellen wat ik moet leren uit de leeractiviteiten.
