# Supplementary material for: The validity and reliability of the Dutch version of the Student Satisfaction and Self-Confidence in Learning Scale (SCLC) for pharmacy technicians
Source: PLoS One. 2025 Sep 29;20(9):e0331115. doi: 10.1371/journal.pone.0331115 (PMC12478918; doi:10.1371/journal.pone.0331115)
Supplement: Attachment — Submitted filename: Response to reviewers.docx [file pone.0331115.s011.docx]

Reactions to the comments of the reviewer and editor

**We would like to thank the reviewers and the academic editor for their constructive feedback and valuable suggestions, which have helped us to improve the quality and clarity of our manuscript.
Please find in the table below our point-by-point responses to each of the reviewers' and editor’s comments, along with a summary of the corresponding revisions made to the manuscript.**

| Reviewer/editor comment | Author’s response | Location in Revised Manuscript |
| --- | --- | --- |
| Reviewer #1  ‘’THIS IS NOT noval work AND authors used old data. references must be added of 2025. there are no figures and diagrams presented in the manuscript.’’ | While we understand the concern regarding novelty, we respectfully disagree. To our knowledge, this is the first validation study of the SCLC instrument in the Dutch language, specifically tailored to pharmacy technicians. We were unable to identify any prior peer-reviewed publication that has translated, culturally adapted, and validated the SCLC in this context.  We believe this validation is valuable for both the Dutch-speaking educational community and for international researchers aiming to compare SCLC outcomes across linguistic and professional groups. As such, this work aligns with PLOS ONE’s mission to publish methodologically sound research, including studies that contribute to instrument validation and psychometrics, even if the original instrument is not newly developed.  Additionally, we have now clearly indicated in the main text where each table and figure should be placed, in line with PLOS ONE formatting guidelines. We have also updated the references to include recent literature from 2025, as requested. | **Added references:**  1. Student satisfaction and self confidence in learning scale (SSCL)—reliability and validity test of the Polish version S. Katarzyna, Z. Danuta and Z. Jakub Signa Vitae 2023 Vol. 19 Issue 5 Pages 104-111  2. Development and validation of a questionnaire to assess quality of life in Australian adults with type 1 diabetes: a pilot psychometric study J. Paul, C. R. Knight-Agarwal, M. A. Saleh, P. Davoren and R. Jani Scientific Reports 2025 Vol. 15 Issue 1 Pages 22462  3. Translation, Cross-Cultural Adaptation, and Psychometric Validation of the Health Information Technology Usability Evaluation Scale in China: Instrument Validation Study  R. Guo, Z. Zheng, F. Yang and Y. Wu  J Med Internet Res 2025 Vol. 27 Pages e67948 |
| Reviewer #2  1. The authors used Cronbach’s alpha (CA) for assessing reliability and CFA-loading factor for validity. CA assumes a tau-equivalent measurement model in which factor loadings are equal and the measurement errors are not correlated. These contrast with the CFA results. Thus, the CA use is invalid. I suggest that the authors further use the CFA results for deriving composite reliability (CR) or construct reliability index. The authors may refer to Hair et al. (2019). | We thank the reviewer for this valuable comment. The reviewer rightly notes that Cronbach’s alpha assumes tau-equivalence and uncorrelated errors, which is inconsistent with the CFA results. Initially, we based our reliability analysis on previous validation studies of the SCLC, which commonly reported Cronbach’s alpha. However, we agree that calculating Composite Reliability (CR) is a more robust approach, particularly in the context of CFA. Therefore, we have now included CR (and AVE) in the Results section of the manuscript. In the Methods section, we have also added a theoretical explanation of why CR is preferred over Cronbach’s alpha when factor loadings are not equal and error terms may correlate (Hair et al., 2019). |  |
| 2. The authors calculated Cronbach’s alpha of total questionnaire. This is meaningless, since a reliability index is associated only to one latent construct, dimension, or subdimension. | The reviewer is correct that Cronbach’s alpha should only be calculated for a single latent construct, and not for the total questionnaire across multiple dimensions. Following this recommendation, we have removed all references to Cronbach’s alpha from the manuscript to ensure that the reported reliability measures are conceptually appropriate. | The Methods and Results section is adjusted. The table that indicated the Cronbach’s Alpha is deleted. |
| 3. The authors treated the Likert scale data as continuous normal data. Likert scale data has a limited number of distinct values. Naturally, it is ordinal data. And by the equidistance assumption, it can be upgraded into discrete interval data which is not a normal data. To improve the validity of the analysis, I suggest the author to use polychoric correlation matrix supplemented by its asymptotic covariance matrix as the inputs and robust maximum likelihood as the estimation method and corrected chi-square statistics for non-normality in the CFA analysis. | The reviewer suggests that it is inappropriate to use likert scales as interval (continuous) data. However, in a study by  Carifo & Perla (2008) and Sullivan (2013) they have discussed the two views on likert scale: is it ordinal or interval. They specifically state that if you have more than one single likert scale item it is appropriate to use parametric statistics.  “*Likert scales and to use these correlations as the basis for various multivariate analytical techniques, such as multiple regression,* ***factor analysis*** *and meta-analysis, to obtain more powerful and nuanced analyses of the data and research hypotheses being investigated.* (Carifo & Perla, 2008, p1151)”.  For this reason, we deem our current analysis appropriate and reflecting reasonably well what we conclude about the data. However, we are aware that the fit of the model is moderate. For this reason, we have now also provided the fit indices of the model where we show the full model (13 items) as well as the 11-item questionnaire, where we have dropped two items from the SCLC questionnaire (item 10 and 13). This did not result in a better fit than the full model.  For this reason, we have toned down our conclusion that our model show moderate fitness and further research is needed to refine the instrument and improve its model fit.    References   1. Carifio, J., & Perla, R. (2008). Resolving the 50-year debate around using and misusing Likert scales. *Medical education*, *42*(12), 1150-1152. 2. Sullivan GM, Artino AR Jr. Analyzing and interpreting data from likert-type scales. J Grad Med Educ. 2013 Dec;5(4):541-2. |  |
| 4. The reported main fit indices indicate an insufficient model fit. I suggest the authors to accommodate modification indices higher than 5 or 6 instead of 20 step-wisely. Report the CFA results after the estimates provide a good model fit. Please refer for Hair et al. (2019) for the criteria of a good model fit. | Your comment has two components. First, accommodating modification indices with different thresholds. The SCLC questionnaire is a well-validated (in different contexts and in different languages). We aimed to validate whether the translated version also provide a (relatively) well fit with the underlying structure. This structure is imposed by the theoretical framework. In the analysis phase, we dropped the two items and found no significant increasing fit. This made us believe that the fit is moderate at best, in contrast to better fit found in other validation studies. We have adjusted the conclusion accordingly to make this more clear.   Additionally, we have followed your advice for modifying indices higher than 5 and then 6 and found a better model fit. Thus, we have reported our results as such and have explained the theoretical background in the methods section.  Lastly, we double checked the goodness-of-fit indices important for reporting in CFAs and to our knowledge we have reported the appropriate goodness of fit indices. | We adjusted the Methods section and Results section. We have also adjusted our discussion and conclusion accordingly. |
| Editor   1. The RMSEA value of 0.099 exceeds the commonly accepted threshold for good model fit (<0.08). This raises concerns about the robustness of the confirmatory factor analysis model, especially given the small sample size (N=129). | Indeed, the present study shows that our validation attempt is less successful. Also dropping two items did not lead to a better fit. For this reason, we have discussed the limited sample size in the discussion and have adjusted the conclusion. |  |
| 2. The manuscript fails to clearly indicate where Table 1, Table 2, Table 3, Table 4, and Figure 1 should be inserted in the main text. | We have adjusted the Tables and Figures according to the PLOS ONE guidelines. |  |
| 3. The authors should explain in advance the criteria used for considering item deletion. | We have explained this. | See Method section |
| 4. The terms like "instructor" and "teacher" may be interpreted differently in digital learning. This ambiguity could be mitigated by clarifying in the methods or providing alternative wording tested during pilot phases. | We have added the explanation of these terms.  We aimed to validate a questionnaire that could be used among pharmacy technicians across multiple educational settings, including both online and in-person learning environments. However, it is possible that the pharmacy technicians’ interpretation of certain terms led to some ambiguity in how they understood these items. We acknowledge this and have addressed and explained this issue in the Discussion section of the manuscript. | See Discussion section. |
